# IGF-1 Signaling Regulates Mitochondrial Remodeling during Myogenic Differentiation

**DOI:** 10.3390/nu14061249

**Published:** 2022-03-16

**Authors:** Xin Guan, Qiyang Yan, Dandan Wang, Guocheng Du, Jingwen Zhou

**Affiliations:** 1 Science Center for Future Foods, Jiangnan University, Wuxi 214122, China; guanxin@jiangnan.edu.cn (X.G.); 6200201094@stu.jiangnan.edu.cn (Q.Y.); beyondan_good@163.com (D.W.); gcdu@jiangnan.edu.cn (G.D.); 2 Engineering Research Center of Ministry of Education on Food Synthetic Biotechnology, Jiangnan University, Wuxi 214122, China; 3 Jiangsu Province Engineering Research Center of Food Synthetic Biotechnology, Jiangnan University, Wuxi 214122, China

**Keywords:** IGF-1, energy metabolism, muscle regeneration, myogenic differentiation, mitochondrial remodeling, mitochondrial biogenesis, mitophagy

## Abstract

Skeletal muscle is essential for locomotion, metabolism, and protein homeostasis in the body. Mitochondria have been considered as a key target to regulate metabolic switch during myo-genesis. The insulin-like growth factor 1 (IGF-1) signaling through the AKT/mammalian target of rapamycin (mTOR) pathway has a well-documented role in promoting muscle growth and regeneration, but whether it is involved in mitochondrial behavior and function remains un-examined. In this study, we investigated the effect of IGF-1 signaling on mitochondrial remodeling during myogenic differentiation. The results demonstrated that IGF-1 signaling stimulated mitochondrial biogenesis by increasing mitochondrial DNA copy number and expression of genes such as *Cox7a1*, *Tfb1m*, and *Ppargc1a*. Moreover, the level of mitophagy in differentiating myoblasts elevated significantly with IGF-1 treatment, which contributed to mitochondrial turnover. Peroxisome proliferator-activated receptor gamma coactivator 1-alpha (PGC-1α) and BCL2/adenovirus E1B 19 kDa protein-interacting protein 3 (BNIP3) were identified as two key mediators of IGF-1-induced mitochondrial biogenesis and mitophagy, respectively. In addition, IGF-1 supplementation could alleviate impaired myoblast differentiation caused by mitophagy deficiency, as evidenced by increased fusion index and myosin heavy chain expression. These findings provide new insights into the role of IGF-1 signaling and suggest that IGF-1 signaling can serve as a target for the research and development of drugs and nutrients that support muscle growth and regeneration.

## 1. Introduction

Skeletal muscle is the biggest organ that accounts for about 40–50% of weight in healthy individuals. Except for locomotive control, skeletal muscle is the major tissue for digesting and utilizing glucose, as well as the largest protein reservoir in the body [1]. Thus, it comes as no surprise that excessive muscle loss (muscle atrophy) is an important clinical feature in many diseases such as cancer, chronic liver and lung diseases, spinal cord disease, and infections [2,3]. The existence of muscle stem cells endows skeletal muscle the capacity of regeneration, so stimulation of postnatal muscle regeneration has become an important strategy to treat atrophying muscle [4].

Muscle regeneration can be described as a highly coordinated process that begins with activation and proliferation of myoblasts, and functional expression of myogenic regulatory factors, to the fusion of mononuclear myoblasts, ultimately forming multinuclear and contractive muscle fibers [5,6]. Increasing reports and reviews have emphasized mitochondria to be positively involved in the muscle regeneration process [7,8]. Oxidative phosphorylation-active myotubes are generated from mostly glycolytic myoblasts during myogenesis, indicating that the energy metabolism center of cells—mitochondria—has changed significantly in the activity and function [9]. Because mitochondria are dynamic organelles, during myogenesis a brand-new and qualified mitochondrial network is formed along with successive mitochondrial fission, fusion, mitophagy, and biogenesis [10]. The influence of mitochondrial dynamics and function on myogenesis and muscle disease have been investigated and revealed to some extent [11,12]. 

Stimulation of mitochondrial biogenesis can promote the myogenic process and repair atrophying muscle tissues [13,14]. Peroxisome proliferator-activated receptor gamma coactivator-1 alpha (PGC-1α) is a key regulator of mitochondrial biogenesis, which plays an essential role in regulating the transcription of nuclear genes that encode mitochondrial proteins [15,16]. Numerous studies have demonstrated that stimulation of PGC-1α expression can improve muscle mass and alleviate atrophy [13,17]. To deal with the stress and promote survival during myogenic differentiation, cellular protein and organelles were degradative via autophagy. It has been reported that autophagy played a critical role in regulating the mitochondrial network during myogenesis [18]. Mitophagy is a degradative process specific for the removal of damaged or aged mitochondria and its essential role in the myogenic differentiation process is increasingly highlighted [10,14]. An intact mitophagic flux requires the collaboration of multiple proteins including mitochondrial membrane proteins such as PTEN-induced putative kinase 1 (PINK1) and BCL2/adenovirus E1B 19 kDa protein-interacting protein 3 (BNIP3), cargo proteins like SQSTM1/p62 (p62), phagophore proteins such as ATGs complex and LC-3I/II, and lysosome-related proteins [19]. Disruption of the mitophagic flux through knockdown of ATG5, ATG7, BNIP3, or p62 has been proved to impair the myogenic differentiation and myogenesis dramatically [20,21].

Throughout previously reported chemical regulators and nutrient supplements that promote muscle growth, activation of the insulin/insulin-like growth factor-1 (IGF-1) signaling is no doubt one of the most important underlying mechanisms [22]. In the body, IGF-1 is mainly secreted by the liver and skeletal muscle, and the intrinsic IGF-1 secretion by muscle cells is especially important for activating muscle response signaling [23]. In addition, resistance exercise can induce the secretion and utilization of IGF-1 in muscle, contributing to its reputation as a recognized way for muscle growth [24]. The effect of IGF-1 is generally attributed to activation of anabolic signaling such as RAS/MAPK and PI3K/AKT pathways, which enhance protein synthesis and reduce protein degradation [24]. Recently, the administration of IGF-1 has been observed to have beneficial effects on restoring mitochondrial functions and treating diseases related to mitochondrial dysfunction [25]. Besides, IGF-1 is linked to regulating mitochondrial biogenesis and mitophagy, which promote mitochondrial protection in normal liver tissue and breast cancer cells [26]. However, the role of IGF-1 signaling in regulating mitochondrial dynamics and function during muscle regeneration remains largely unexamined. 

Here, we explored the effect of IGF-1 signaling on mitochondrial behavior and function in the myoblast differentiation process. We found that IGF-1 signaling regulated both mitochondrial biogenesis and mitophagy in differentiating myoblasts. Inhibition of IGF-1 signaling led to mitochondrial dysfunction and differentiation failure. Moreover, activation of IGF-1 signaling alleviated impaired myoblast differentiation caused by mitochondrial function deficit. 

## 2. Materials and Methods

### 2.1. Chemicals and Reagents

C2C12 myoblasts were purchased from ATCC (Shanghai, China). Dulbecco modified Eagle’s medium (DMEM), penicillin/streptomycin (P/S), fetal bovine serum (FBS), and horse serum (HS) were purchased from Thermo Scientific (Waltham, MA, USA). IGF-1, BMS754807 (BMS), 3-MA, and chloroquine (CQ) were purchased from MedChemExpress (Shanghai, China). Antibodies such as anti-phospho-IGF-1R, anti-IGF-1R, anti-phospho-AKT, anti-AKT, anti-phospho-mTOR, and anti-mTOR were obtained from Cell Signaling Technology (Danvers, MA, USA). Anti-BNIP3, anti-myosin heavy chain (MHC), anti-Myogenin (MyoG), and anti-TOMM20 were obtained from Santa Cruz Biotechnology (Santa Cruz, CA, USA). Antibodies such as anti-α-tubulin, anti-GAPDH, anti-PGC-1α, anti-p62, and anti-LC3 were obtained from Protientech (Wuhan, China).

### 2.2. Cell Culture

C2C12 cells were cultured in the growth medium consisting of DMEM, 10% heat-inactivated FBS, and 5 mg/mL P/S. To induce myogenic differentiation, the culture medium was changed to the basal differentiation medium supplemented with IGF-1, BMS, or vehicle DMSO when C2C12 cells were grown to 90% confluence and continued to culture for 3 to 5 days. The basal differentiation medium (DM) was DMEM plus 2% heat-inactivated HS and 5 mg/mL P/S. Primary porcine satellite cells were isolated from large white pigs as reported previously and cultured on collagen-coated plates [27]. The culture medium and procedures for satellite cells were the same as C2C12 cells.

### 2.3. SiRNA Transfection

SiRNAs targeting BNIP3 (antisense: 5′-AAUCCUCAUCCUGCAAAGCAG-3′; sense: 5′-GCUUU GCAGGAUGAGGAUUTT-3′), PGC-1α (antisense: 5′-UCUACACCACUUCAAUCCACCTT-3′; sense: 5′-GGUGGAUUGAAGUGGUGUAGATT-3′), and negative siRNA were synthesized in Genewiz, Suzhou, China. Cells were transfected with siRNAs (10 nM) using Lipofectamine RNAiMAX (Thermo Scientific) according to manufacturer’s instructions.

### 2.4. Immunofluostaining

Cells were permeabilized with 0.5% Triton X-100 and blocked with 1% BSA for 1 h, then incubated with anti-MHC primary antibody at 4 °C overnight and subsequently with CoraLite488-conjugated secondary antibodies (Proteintech, Wuhan, China) at room temperature for 1 h. Images were captured with a fluorescence microscope (MF52-M, Mshot, Guangzhou, China) and the MHC-positive myotubes were calculated to quantify the differentiation efficiency. The fusion index was determined by dividing the number of nuclei in MHC-positive cells and by total number of nuclei in each image [27]. In some experiments that analyze mitophagy, cells were infected with an adenovirus encoding GFP-LC3 (Beyotime, Shagnhai, China) to visualize autophagosomes 48 h prior to permeabilization and the anti-TOMM20 antibody was used to label mitochondria. Then, images were captured with a Nikon A1 laser scanning confocal microscope (Nikon, Tokyo, Japan). Mitophagy was assessed by calculating the colocalization between GFP-LC3-positive autophagosomes and TOMM20-labeled mitochondria [28]. At least three images were analyzed for each experimental sample using the ImageJ software. 

### 2.5. Western Blotting

Cells were lysed with RIPA lysis buffer (50 mM Tris, 150 mM NaCl, 0.1% SDS, 1% sodium deoxycholate (Beyotime, Shagnhai, China) for 30 min on ice and total protein lysates were measured by the BCA method (Beyotime, Shagnhai, China). Proteins were separated by SDS-PAGE and transferred onto PVDF membranes. After blocking with BSA containing 0.05% Tween 20 for 1 h at room temperature, PVDF membranes were incubated with primary antibodies overnight at 4 °C and subsequently with HRP-labeled secondary antibodies (Beyotime, Shagnhai, China) for 1 h at room temperature. A Tanon 4600SF scanner system was used for protein detection (Tanon, Shagnhai, China). The band density was analyzed using ImageJ software with α-tubulin or GAPDH as internal control, then normalized to vehicle control (NC).

### 2.6. Transmission Electron Microscopy (TEM)

Cells were fixed in 2.5% glutaraldehyde (pH 7.4) for 2 h and embedded in agarose with low melting point. After washing with 0.1M PBS and fixing in 1% osmic acid at 4 °C for 2 h, the samples were dehydrated in a graded series of ethanol. Subsequently, the samples were embedded in Epon-Araldite resin for penetration and placed in a model for polymerization. After sectioning blocks, ultrathin sections were counterstained with uranyl acetate and lead citrate, and then observed with a HT7800 transmission electron microscope (HITACHI, Tokyo, Japan).

### 2.7. Quantitative Real-Time PCR (RT-PCR)

RNA extraction was carried out using the Tatal RNA Kit I R6834 (Omega Bio-tek, Norcross, GA, USA) as per the manufacturer’s instructions. cDNA synthesis was performed using the HiScript II Q Select RT SuperMix (Vazyme, Nanjing, China) and the RT-PCR was carried out with the ChamQ Universal SYBR qPCR Master Kit (Vazyme) as per the manufacturer’s instructions. The fold change of gene expression was normalized with 18S ribosomal RNA and relative fold change was calculated using the 2^−ΔΔCT^ method. All primer sequences used for RT-PCR are listed in Table 1.

### 2.8. Flow Cytometry

To measure the mitochondrial ROS level, cells were incubated with 5 μM of MitoSOX Red (Invitrogen, Carlsbad, CA, USA) at 37 °C for 30 min, washed twice with PBS, and resuspended in HBSS. The fluorescence of MitoSOX Red was analyzed via flow cytometry with excitation at 488 nm and emission at 620 nm. The median fluorescence intensity from 10,000 cells of individual samples were calculated and normalized to the Day 0 samples. To analyze the mitochondrial mass, cells were incubated and stained with Mitotracker Green (MTG) at 37 °C for 30 min, washed twice with PBS, and resuspended in HBSS. The fluorescence of MTG was analyzed via flow cytometry with excitation at 488 nm and emission at 516 nm. The median fluorescence intensity from 10,000 cells of individual samples were calculated and normalized to the Day 0 samples. To analyze the mitochondrial membrane potential, cells were incubated with the 5,5′,6,6′-tetrachloro-1,1′-3,3′-tetraethyl-benzimidazolylcarbocyanine iodide (JC-1) probe at 37 °C for 20 min, washed twice with PBS, and resuspended in HBSS. To analyze the apoptosis, total cells were collected and stained with the FITC-Annexin V/PI detection kit (BD Biosciences, San Jose, CA, USA) for 30 min, washed twice with PBS, and resuspended in the Annexin V binding buffer. All flow cytometry analyses were performed on a FACSCalibur with the Cellquest Pro software (BD Biosciences).

### 2.9. Statistical Analysis

Experiments were conducted at least three times independently. Quantitative data are presented as the mean ± standard deviation (SD). The statistical significance was calculated using Student’s unpaired *t*-test. Values of *p* < 0.05 were considered statistically significant.

## 3. Results

### 3.1. IGF-1 Signaling Is Essential for Myogenic Differentiation

**Figure 1:**
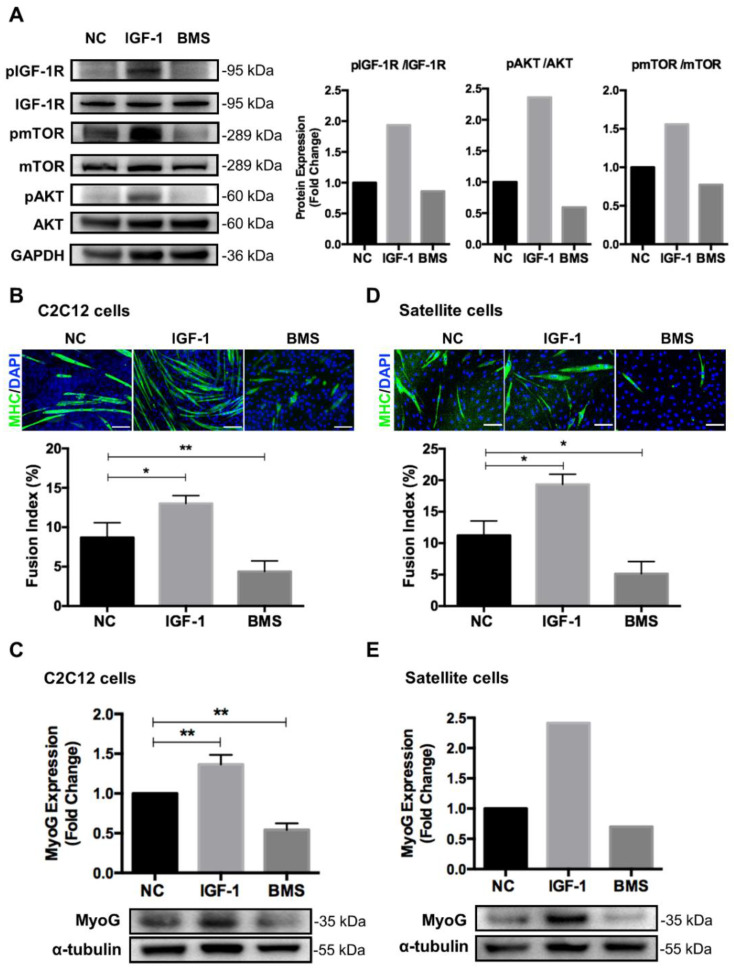
IGF-1 signaling regulates myoblast differentiation. C2C12 myoblasts or satellite cells were cultured in the DM supplemented with 0.1% DMSO, 10 ng/mL IGF-1, or 200 nM BMS for 5 days. (**A**) Representative immunoblots of IGF-1R, pIGF-1R, mTOR, pmTOR, AKT, pAKT, or GAPDH in C2C12 cells treated with 0.1% DMSO (NC), 10 ng/mL IGF-1, or 200 nM BMS. (**B**) Representative images of myotube formation and quantitative analysis of fusion index in C2C12 cells. Cells were stained with DAPI (blue) and myosin heavy chain (MHC, green) to visualize nuclei and myosin, respectively. Scale bar = 100 μm. (**C**) Representative immunoblots and quantitative analysis of MyoG and α-tubulin in C2C12 myotubes. (**D**) Representative images of myotube formation and quantitative analysis of fusion index in satellite cells. (**E**) Representative immunoblots and quantitative analysis of MyoG and α-tubulin in satellite cells. Data represent means ± SD. * *p* < 0.05 and ** *p* < 0.01. Abbreviations: IGF-1, Insulin-like growth factor 1; BMS, BMS754807; NC, vehicle control; mTOR, mammalian target of rapamycin; AKT, protein kinase B; GAPDH, glyceraldehyde-3-phosphate dehydrogenase; MyoG, myogenin.

C2C12 myoblasts are a classical model that has been widely used to study muscle growth and regeneration mechanisms at the cellular level [29]. Muscle development has been reported to be dependent on the engagement of multiple hormones and cytokines [30]. First, we determined the effect of IGF-1 signaling on myogenesis by enhancing or inhibiting IGF-1 signaling in myoblasts. C2C12 myoblasts were cultured in the DM supplemented with 0.1% DMSO, 10 ng/mL IGF-1, and 200 nM BMS (an IGF-1R tyrosine kinase inhibitor) [31], respectively, to induce differentiation for 5 days. Western blotting assays showed that IGF-1 increased the phosphorylation levels of IGF-1R, Akt, and mTOR, indicating the activation of IGF-1 signaling. BMS inhibited the activation of small amounts of endogenous IGF-1R, Akt, and mTOR (Figure 1A). Stimulation of C2C12 cells with IGF-1 generated more multinucleated myotubes, but the number of myotubes declined dramatically when cells were treated with BMS. We then quantified the differentiation efficiency based on the number of nuclei in MHC-positive myotubes. After 5 days of differentiation, the fusion index increased about two times when exposing C2C12 myoblasts to 10 ng/mL IGF-1 but decreased about two times upon treatment with 200 nM BMS (Figure 1B). The protein expression of the key myogenic factor MyoG also demonstrated the distinct differentiation capability when activating or inhibiting IGF-1 signaling in myoblasts (Figure 1C). Furthermore, to confirm the effect of IGF-1 signaling on myogenic differentiation of primary satellite cells, satellite cells were isolated from porcine muscle tissues and induced to differentiation in the presence of IGF-1 or BMS. Paralleling with the results of C2C12 cells, IGF-1 treatment markedly increased the fusion index and MyoG expression of differentiating satellite cells, while BMS treatment decreased both of them (Figure 1D,E).These results suggested that the IGF-1 signaling played a critical role in regulating myogenic differentiation. 

### 3.2. Inhibition of IGF-1 Signaling Leads to Mitochondrial Dysfunction and Damage

**Figure 2:**
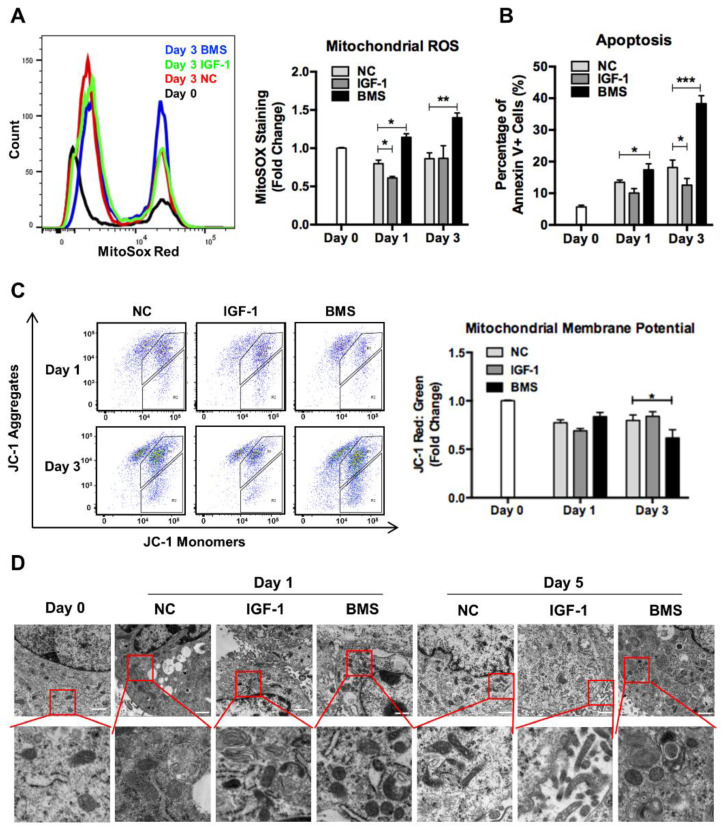
Inhibition of IGF-1 signaling leads to mitochondrial dysfunction and damage. (**A**) Representative flow charts and quantitative analysis of mitochondrial ROS levels in DMSO, IGF-1, and BMS-C2C12 that were stained with MitoSoX cells during differentiation. (**B**) Quantitative analysis of apoptotic (Annexin V+) cells during differentiation. (**C**) Quantitative analysis of the JC-1 red: green fluorescence ratio with FACS during differentiation. (**D**) Electron micrographs of differentiating C2C12 cells. Scale bar = 1 μm. Data represent means ± SD. * *p* < 0.05, ** *p* < 0.01, and *** *p* < 0.001. Abbreviations: ROS, reactive oxygen species; JC-1, 5,5′,6,6′-tetrachloro-1,1′-3,3′-tetraethyl-benzimidazolylcarbocyanine iodide.

Mitochondria are the center of cell energy generation, so the significant switch of energy metabolism from glycolysis to oxidative phosphorylation during differentiation depends on healthy and functional mitochondrial networks [8]. We found that mitochondrial reactive oxygen species (ROS) decreased at day 1 and then increased at day 3 of differentiation in control and IGF-1-treated myoblasts, but cells exposed to BMS exhibited continuously elevated mitochondrial ROS levels during differentiation, indicating the mitochondrial dysfunction (Figure 2A). Then, we measured the mitochondrial membrane potential (MMP) of differentiating myoblasts with JC-1 staining. The loss of MMP could be expressed by a decrease in the ratio of JC-1 aggregates (red fluorescence) to JC-1 monomers (green fluorescence) [32]. Cells in the control and IGF-1 groups showed a reduction in MMP from the beginning of differentiation and then maintained at a stable level. In contrast, the MMP of cells exposed to BMS displayed a slightly higher level than that of the other two groups at day 1 but decreased significantly at day 3 of differentiation (Figure 2C). Moreover, although the number of Annexin V+ cells increased during the differentiation process, BMS-exposed cells exhibited a significantly higher level than the other two groups, suggesting cell apoptosis caused by impaired mitochondria (Figure 2B). Subsequently, we examined the mitochondrial morphology in differentiating cells with TEM. Compared to normal elongated mitochondria in control and IGF-1-treated cells, the mitochondrial structure in BMS-treated cells exhibited a marked alteration, exhibiting numerous donut-shaped or swelling mitochondria (Figure 2D).

### 3.3. IGF-1 Signaling Regulates Mitochondrial Biogenesis during Myogenic Differentiation

**Figure 3:**
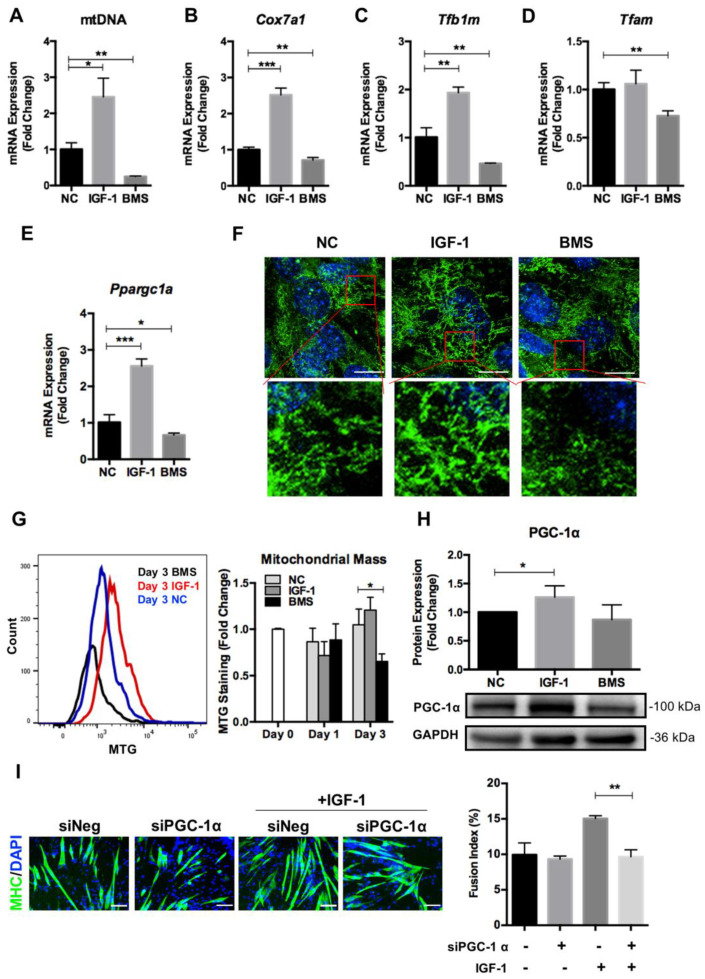
IGF-1 signaling regulates mitochondrial biogenesis during myogenic differentiation. (**A**) Mitochondrial DNA (mtDNA) content in C2C12 myotubes after 3 days of differentiation. (**B**–**E**) mRNA expression of *Cox7a1* (**B**), *Tfb1m* (**C**), *Tfam* (**D**), and *Ppargc1a* (**D**) genes in C2C12 cells treated with DMSO (NC), 10 ng/mL IGF-1, or 200 nM BMS after 3 days of differentiation. (**F**) The Mito Tracker Green (MTG) staining of C2C12 cells after 3 days of differentiation. (**G**) The mitochondrial mass evaluation by MTG staining and quantified by FACS. (**H**) Representative immunoblots and quantitative analysis of PGC-1α and GAPDH in C2C12 cells after 3 days of differentiation. (**I**) Immunofluorescent staining of MHC (green) and DAPI (blue) and quantitative analysis of the fusion index for siNeg and siPGC1α C2C12 cells with or without IGF-1 treatment after 5 days of differentiation. Scale bar = 100 μm. Data represent means ± SD. * *p* < 0.05, ** *p* < 0.01, and *** *p* < 0.001.

Previous reports have proved that mitochondrial remodeling occurs along with cell differentiation, which is mainly regulated through mitochondrial biogenesis and mitophagy [33]. To investigate whether the IGF-1 signaling affected mitochondrial biogenesis during myogenic differentiation, we first evaluated the transcription of mitochondrial-related genes in differentiated C2C12 myoblasts. The mitochondrial DNA (mtDNA) copy number, which was determined by the ratio of *Nd1* to *Lpl* (mtDNA/nuclear DNA), increased significantly in differentiating myoblasts exposed to IGF-1 but decreased markedly in BMS-treated cells (Figure 3A). Besides, the relative expression levels of a series of genes that regulated mitochondrial biosynthesis including *Cox7a1*, *Tfam*, *Tfb1m*, and *Ppargc1a*, were higher in IGF-1-treated cells but lower in BMS-treated cells (Figure 3B–E). The MitoTracker staining in myotubes formed in the presence of IGF-1 showed more linear mitochondria, but much less MitoTracker signaling could be observed in cells exposed to BMS (Figure 3F). Consistently, the FACS analysis for MitoTracker staining exhibited significantly reduced mitochondrial mass in the BMS group (Figure 3G). These results suggested that the IGF-1 signaling was involved in mitochondrial biogenesis during myogenic differentiation.

Next, we tested the effect of IGF-1 signaling on PGC-1α expression as it has been proved to be a key regulator of mitochondrial dynamics and biogenesis in skeletal muscle development [15,34]. Activation of IGF-1 signaling elevated the PGC-1α protein expression significantly, and inhibition of IGF-1 signaling with BMS reduced the PGC-1α level slightly in differentiating myoblasts (Figure 3H). Furthermore, we measured the pro-differentiating effect of IGF-1 after suppressing PGC-1α expression with siRNA in myoblasts. The addition of siPGC-1α impaired the pro-differentiation effect of IGF-1 in myogenic differentiation markedly, as indicated by decreased fusion index (Figure 3I). Taken together, the above data indicated that IGF-1 promoted mitochondrial biogenesis during myogenic differentiation and the PGC-1α expression was required for IGF-1-induced differentiation.

### 3.4. IGF-1 Signaling Regulates Mitophagy during Myogenic Differentiation 

**Figure 4:**
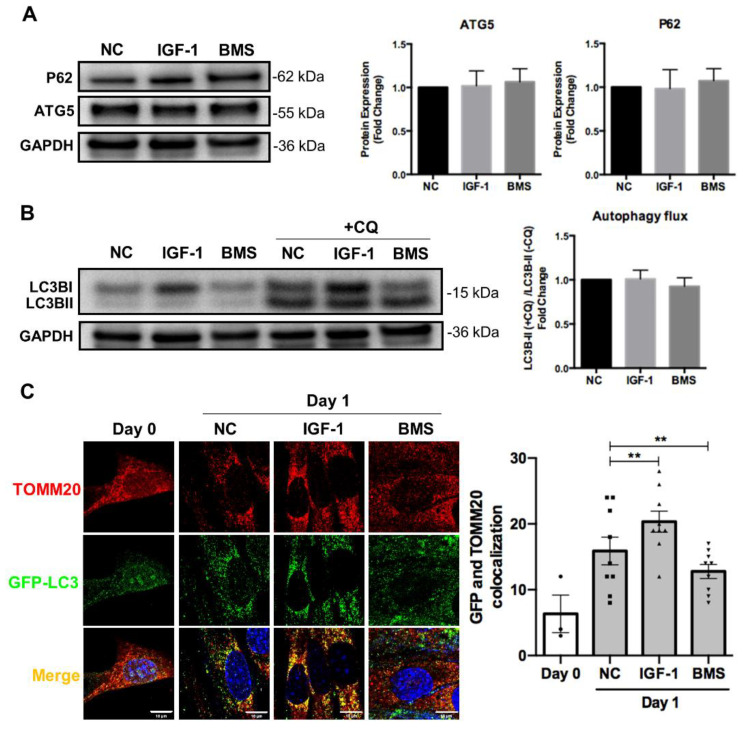

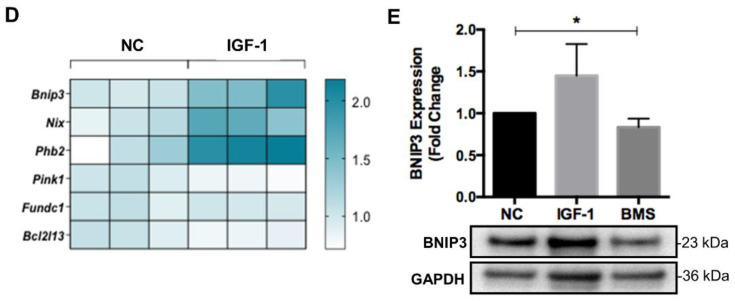
IGF-1 signaling regulates mitophagy during myogenic differentiation. (**A**) Representative immunoblots and quantitative analysis of SQSTM1/p62 (p62), ATG5, and GAPDH in differentiating cells. (**B**) Representative immunoblots and quantitative analysis of LC3B and GAPDH in differentiating cells with or without chloroquine (CQ) treatment. In the experiments for monitoring the autophagic flux, cells were treated with 50 μM CQ for 4 h as CQ treatment leaded to LC3B-II aggregation. The autophagic flux was analyzed through the ratio of LC3B-II expression in CQ-treated and untreated cells. (**C**) Representative fluorescent images and quantitative analysis of colocalization (yellow) in ad-GFP-LC3 (green) transfected cells and stained with anti-TOMM20 (red) at day 1 of differentiation. Scale bar = 10 μm. (**D**) mRNA expression of mitophagy receptors in differentiating C2C12 myoblasts with or without treatment of IGF-1. (**E**) Representative immunoblots and quantitative analysis of BNIP3 and GAPDH in C2C12 cells after 3 days of differentiation. Data represent means ± SD. * *p* < 0.05 and ** *p* < 0.01.

It has been proven that autophagy and mitophagy are both activated during cell differentiation to mediate non-specific and mitochondria-specific degradation, respectively [10,28]. Meanwhile, autophagy and mitophagy also protect cells from oxidative stress and apoptosis during differentiation [19]. Thus, we investigated the effect of IGF-1 signaling on several proteins involved in autophagosome formation and general autophagy. However, we did not observe significant differences in the expression of autophagosome proteins such as p62 and ATG5, as well as LC-3II autophagy flux in myoblasts stimulated by IGF-1 or BMS during differentiation (Figure 4A,B). Then, we examined the level of mitophagy by assessing the co-localization between GFP-LC3 positive autophagosomes and TOMM20-labeled mitochondria. As shown in Figure 4C, elevating IGF-1 signaling led to a significantly higher number of TOMM20 signals that co-localized with GFP-LC3-positive autophagosomes after 1 day of differentiation. In contrast, reduced co-localization between autophagosomes and mitochondria could be observed when inhibiting the IGF-1 signaling by BMS during differentiation. It has been proposed that two kinds of canonical mitophagy systems mediated the mitochondrial clearance, specifically, Pink1/Parkin-dependent mitophagy and ubiquitin-independent mitophagy [35]. Therefore, we next measured the expression levels of genes of *Pink1* and other ubiquitin independent mitophagy receptors including *Bnip3*, *Nix*, *Phb2*, *Fundc1*, and *Bcl2l13* via mRNA measurement. IGF-1 supplement increased the mRNA expression levels of *Bnip3*, *Nix*, and *Phb2*, but did not affect the expression of *Pink1*, *Fundc1*, and *Bcl2l13* (Figure 4D). Previous report demonstrated that myoblasts were failed to form myotubes after BNIP3 knockout, indicating the significant role of BNIP3 in myogenesis [19]. Therefore, we further investigated the effect of IGF-1 signaling on BNIP3 expression via Western blot assays. The results confirmed the elevated expression of BNIP3 protein after activating the IGF-1 signaling but reduced BNIP3 expression upon suppression (Figure 4E). These data indicated that IGF-1 signaling regulated BNIP3-mediated mitophagy during myogenic differentiation.

### 3.5. IGF-1 Induces PGC-1α and BNIP3 Accumulation Dose-Dependently during Differentiation

**Figure 5:**
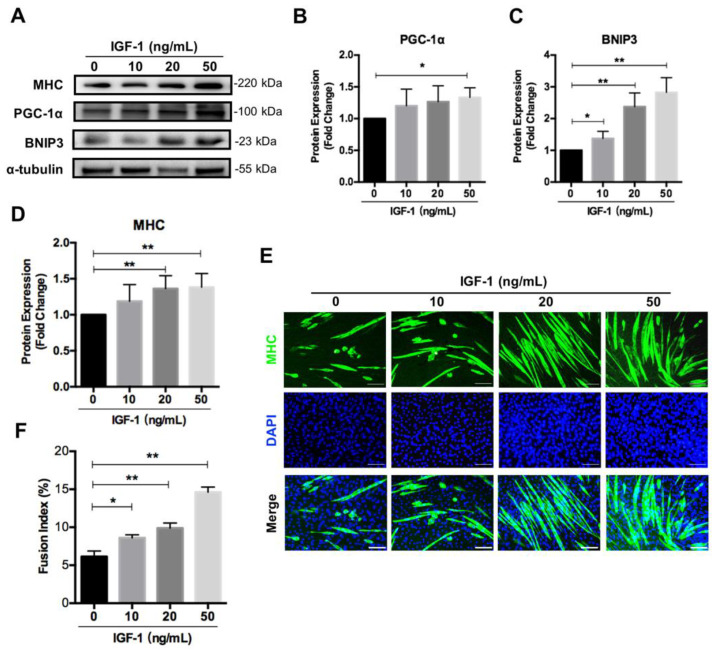
IGF-1 induces PGC-1α and BNIP3 accumulation dose-dependently. (**A**–**D**) Representative immunoblots and quantitative analysis of PGC-1α (**B**), BNIP3 (**C**), MHC (**D**), and α-tubulin in C2C12 cells after 5 days of differentiation. (**E**) Immunofluorescent staining of MHC (green) and DAPI (blue) of the fusion index in C2C12 cells treated with 0~50 ng/mL of IGF-1 after 5 days of differentiation. (**F**) Quantitative analysis of the fusion index. Scale bar = 100 μm. Data represent means ± SD. * *p* < 0.05 and ** *p* < 0.01.

Since IGF-1 signaling affected mitochondrial function and regulated mitochondrial remodeling through mitochondrial biogenesis and mitophagy during myogenic differentiation, we asked whether there was a dose–response relationship between IGF-1-promoted myogenic differentiation and mitochondrial biogenesis and mitophagy. C2C12 myoblasts were exposed to various concentrations of IGF-1 and the differentiation efficiency was measured, as well as the expression levels of mitochondrial biogenesis marker PGC-1α and mitophagy marker BNIP3. As expected, the expression of PGC-1α and BNIP3 increased dose-dependently when treating C2C12 cells with IGF-1 from 0 ng/mL to 50 ng/mL (Figure 5A–C). At the same time, the myogenic differentiation efficiency generally enhanced with the increase of IGF-1 concentration, as evidenced by quantitating analyses of MHC immunostaining and Western blotting (Figure 5A,D–F). These results confirmed the effect of IGF-1 on mediating PGC-1α and BNIP3 expression during myogenic differentiation.

### 3.6. IGF-1 Alleviates Impaired Myoblast Differentiation Caused by Mitophagy Deficiency

**Figure 6:**
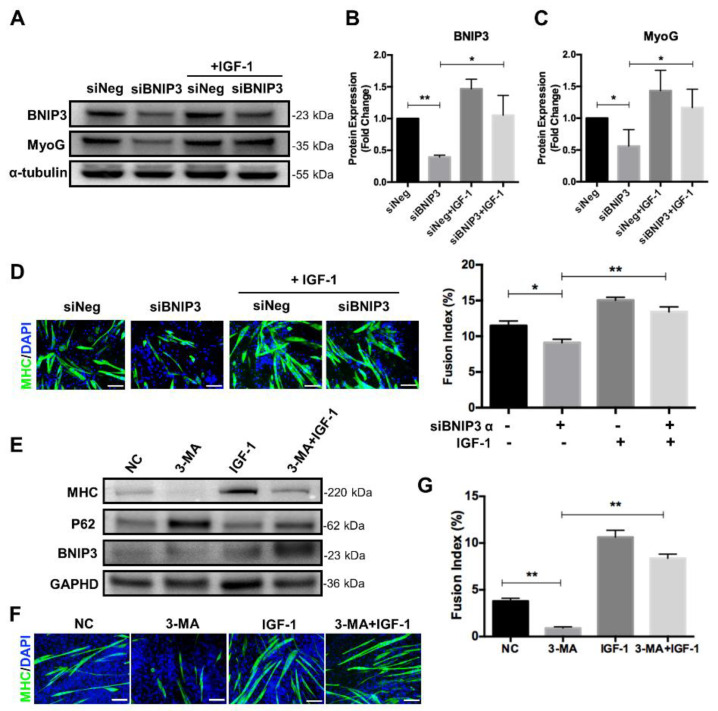
IGF-1 increases the differentiation potential in mitophagy- and autophagy-deficient myoblasts. (**A**–**C**) Representative immunoblots and quantitative analysis of BNIP3, MyoG, and α-tubulin in C2C12 cells after 5 days of differentiation. (**D**) Immunofluorescent staining of MHC (green) and DAPI (blue) and quantitative analysis of the fusion index for siNeg and siBNIP3 C2C12 cells with or without IGF-1 treatment after 5 days of differentiation. (**E**) Representative immunoblots analysis of MHC, p62, BNIP3, and GAPDH in C2C12 cells after 5 days of differentiation in the presence of 3-MA and/or IGF-1. (**F**) Immunofluorescent staining of MHC (green) and DAPI (blue) of differentiating C2C12 cells with treatment of 3-MA and/or IGF-1. (**G**) Quantitative analysis of the fusion index. Scale bar = 100 μm. Data represent means ± SD. * *p* < 0.05 and ** *p* < 0.01.

To further confirm the effect of IGF-1 signaling in regulating mitochondrial mitophagy during myogenic differentiation, we produced BNIP3-suppressed myoblasts with specific siRNA (Figure 6A,B). Consistent with the previous report, myogenic differentiation was impaired upon suppression of BNIP3, as demonstrated by decreased fusion index and MyoG expression (Figure 6A,C,D). In contrast, differential inhibition was relieved by activation of the IGF-1 signaling during the differentiation process. The addition of IGF-1 increased the expression level of BNIP3 and thus elevated the differentiation potential, as evidenced by enhanced MyoG expression (Figure 6A,C). Consistently, the fusion index was increased in BNIP3-suppressed myoblasts upon the addition of IGF-1 (Figure 6D). These results suggested that IGF-1 supplementation could alleviate differentiation impairment caused by BNIP3 inhibition.

It has been reported that inhibition of autophagy with 3-MA impaired myoblast differentiation, so we wondered whether stimulation of IGF-1 signaling could promote differentiation in autophagy-deficient myoblasts. C2C12 myoblasts were cultured in the DM supplemented with 1 mM 3-MA, 10 ng/mL IGF-1, or 3-MA plus IGF-1 and induced to differentiation for 5 days. As shown in Figure 6E, the addition of 3-MA resulted in a dramatic accumulation of p62, indicating inhibition of autophagy. At the same time, the differentiation potential of 3-MA-treated cells was weakened significantly, as demonstrated by decreased expression of MHC and lack of multinucleated and MHC positive myotubes (Figure 6E,F). Interestingly, compared to 3-MA treatment, combined addition of IGF-1 and 3-MA during differentiation reduced p62 accumulation and elevated the expression of MHC (Figure 6E). Meanwhile, more multinucleated myotubes and increased fusion index could be observed after 5 days of differentiation (Figure 6F,G). Collectively, these data suggested that activation of IGF-1 signaling could promote mitophagy and autophagy flux and thus improve myogenic differentiation.

## 4. Discussion

Myogenic differentiation is an important process in muscle development and regeneration, which transforms immature myoblasts into mature and contractile muscle fibers. The important role of autophagy and mitophagy in regulating myogenic differentiation has been reported previously [16,19]. Here, we provide strong evidence to support the role of IGF-1 signaling in maintaining mitochondrial function and regulating mitochondrial biogenesis and mitophagy during differentiation. Furthermore, we found that IGF-1 dose-dependently induced the expression of PGC-1a and BNIP3, which are key mediators of mitochondrial biogenesis and mitophagy, respectively.

Mitochondria are essential organelles of muscle cells, which govern oxidative phosphorylation to generate ATP and regulate calcium homeostasis, as well as oxidative stress and apoptosis signaling. Many studies have revealed the importance of healthy mitochondrial networks in supporting myogenesis and maintaining skeletal muscle function [36,37]. Previously, IGF-1 has been shown supportive for mitochondrial homeostasis and applied to treat mitochondrial dysfunction-related diseases [25]. In this study, activation of IGF-1 signaling in myoblasts promoted myogenic differentiation, accompanied by increased expression of mitochondrial genes and proteins. In contrast, myoblasts exposed to IGF-1R kinase inhibitor failed to form myotubes and exhibited mitochondrial dysfunction, as evidenced by increased mitochondrial ROS, reduced MMP, elevated apoptosis, and abnormal mitochondrial morphology. Although IGF signaling has been linked previously to induce muscle hypertrophy via protein synthesis pathways and block muscle atrophy via the ubiquitin-ligases MuRF1 and MAFbx [24], our study provides new sights for IGF-1 signaling from the perspective of regulating mitochondrial remodeling.

To meet the increased energetic demand of myotubes, the mitochondrial mass is increased markedly through mitochondrial biogenesis. PGC-1α is a transcriptional coactivator that can co-activate hormone nuclear receptor PPARs and nuclear respiratory factors NRF-1 and NRF-2, which regulate the transcription of a series of mitochondrial transporters and transcription factors like TFAM and TFBM [38]. Therefore, PGC-1α has been considered to be one key inducer of mitochondrial biogenesis. In our study, it has been observed that stimulation of the IGF-1 signaling induced a significant increase in PGC-1α expression and elevated COX7A1, TFB1M, and mtDNA content, thereby increasing mitochondrial function and mass in differentiating myoblasts. Moreover, suppression of PGC-1α blocked enhanced fusion index by IGF-1 markedly, indicating the important role of regulating mitochondrial biogenesis in IGF-1-induced myogenic differentiation. Except for PGC-1α, the PGC-1 family transcriptional co-activators also include PGC-1β and PRC, whose expression varies between tissues and cell types. Similar to our results, Amy Lyons and co-workers demonstrated that in breast cancer cells IGF-1 facilitated mitochondrial biogenesis and potential through induction of PGC-1β and PRC [26]. The mechanism underlying IGF-1 signaling-promoted PGC-1α expression could be attributed to the existence of a yin-yang 1(YY1)/PGC-1α transcriptional complex in muscle tissues and cells. The YY1/PGC-1α complex is required for mitochondrial gene expression and respiration and its transcriptional function is directly modulated by mTOR activity [39]. As IGF-1 is a classic upstream activator of mTOR, it could be deduced that IGF-1 enhanced mitochondrial biogenesis during myogenic differentiation via mTOR/PGC-1α signaling in our work. 

Removal of unnecessary or unadaptable mitochondria is required for the achievement of “metabolic reprogramming” in the stem cell differentiation process, which is known as mitophagy. Our study suggested that the IGF-1 signaling was involved in maintaining the capacity of mitophagy in differentiating myoblasts. More importantly, IGF-1 addition was able to rejuvenate myogenic differentiation in mitophagy or autophagy-deficient myoblasts. Over the recent years, multiple mechanisms have been discovered that mediate mitophagy, mainly including PINK1/Parkin-dependent pathway and LC3-interacting region (LIR) domain-containing receptor-mediated pathways [40]. Here, we found that IGF-1 did not affect the expression of PINK1. Instead, the expression of BNIP3 was associated with IGF-1 signaling activity in differentiating myoblasts. BNIP3 is a Bcl-2 homology 3 (BH3)-only member of the Bcl-2 family that was originally discovered as a pro-apoptotic protein because it can inhibit Bcl-2 proteins [41]. Meanwhile, because of containing the LIR domain, BNIP3 has been proved to interact with LC3II on autophagosomes and mediate mitophagy directly. Recently, Sarah Riis and colleagues reported a conserved Akt/GSK-3β/Nrf2/BNIP3 pathway for regulating mitochondrial morphology and dynamics in mouse embryonic fibroblasts and cancer cells [42], which provides mechanistic clues for IGF-1-dependent BNIP3 transcription during differentiation.

As the world’s population ages and the incidence of cancer increases, treatment aiming at increased muscle growth and regeneration have been given high priority. Skeletal muscle is a highly energy-demanding tissue, where mitochondrial homeostasis and function play a critical role in regulating myogenesis [18]. Some lines of evidence suggest that mitochondrial impairment blocks muscle growth and leads to muscle diseases [37,43]. Therefore, understanding the exact regulatory mechanisms of mitochondrial remodeling during myogenesis is essential to identify effective targets for promoting muscle growth and regeneration. In this study, we observed the beneficial effects of IGF-1 on the improvements in mitochondrial function and turnover during myogenic differentiation by mediating mitochondrial biogenesis and mitophagy. IGF-1 therapy has been proved effective in several aspects including promoting glucose and lipid metabolism, protecting the nervous system, and antioxidants [44], so our findings further highlight IGF-1 as a useful target in treatment aiming muscle regeneration.

## 5. Conclusions

Our study demonstrates that IGF-1 signaling was involved in mitochondrial remodeling during myogenic differentiation. Activation of IGF-1 signaling promoted mitochondrial biogenesis via stimulating PGC-1α and enhanced mitophagy flux via inducing BNIP3, both of which mediated mitochondrial turnover and thus increased myogenic differentiation efficacy. In addition, IGF-1 supplementation could alleviate impaired myoblast differentiation caused by mitochondrial function defects. These findings provide new insights into the role of IGF-1 signaling and highlight the powerful effectiveness of IGF-1 in facilitating muscle regeneration.

## Figures and Tables

**Table 1:** The primer sequences in this study.

Gene	Primer Sequence
*Bcl2l13*	F:5′-ATGGCGTCCTCTACGACTG-3′
	R:5′-GGTGAGGGACCTTGTTGTTTC-3′
*Bnip3*	F:5′-GTTCCAGCCTCCGTCTCTATT-3′
	R:5′-CCTCAGACAGAGTGCTGTTTTTC-3′
*Cox7a1*	F:5′-CAGCGTCATGGTCAGTCTGT-3′
	R:5′-AGAAAACCGTGTGGCAGAGA-3′
*Fundc1*	F:5′-CCCCCTCCCCAAGACTATGAA-3′
	R:5′-CCACCCATTACAATCTGAGTAGC-3′
*Lpl*	F:5′-GAAAGGGCTCTGCCTGAGTT-3′
	R:5′-TAGGGCATCTGAGAGCGAGT-3′
*Nd1*	F:5′-CACTATTCGGAGCTTTACG-3′
	R:5′-TGTTTCTGCTAGGGTTGA-3′
*Nix/Bnip3L*	F:5′-ATGTCTCACTTAGTCGAGCCG-3′
	R:5′-CTCATGCTGTGCATCCAGGA-3′
*Nrf1*	F:5′-GCACCTTTGGAGAATGTGGT-3′
	R:5′-CTGAGCCTGGGTCATTTTGT-3′
*Ppargc1a*	F:5′-GGACATGTGCAGCCAAGACTC-3′
	R:5′-CACTTCAATCCACCCAGAAAGCT-3′
*Phb2*	F:5′- ATCCGTGTTCACCGTGGAAG-3′
	R:5′-CCCGAATGTCATAGATGATGGG-3′
*Pink1*	F:5′-GCTGATCGAGGAGAAGCAG-3′
	R:5′-GATAATCCTCCAGACGGAAGC-3′
*Tfam*	F:5′- CCAAAAAGACCTCGTTCAGC-3′
	R:5′-CTTCAGCCATCTGCTCTTCC-3′
*Tfb1m*	F:5′-CACCGAGGGCTTGGAATGTT-3′
	R:5′-TAGAACCCGCAGCTTTCTGG-3′
*18S*	F:5′-GTAACCCGTTGAACCCCATT-3′
	R:5′-CCATCCAATCGGTAGTAGCG-3′

## Data Availability

The data presented in this study are available on request from the corresponding author.
